# Improved pregnancy outcome in refugees and migrants despite low literacy on the Thai-Burmese border: results of three cross-sectional surveys

**DOI:** 10.1186/1471-2393-11-45

**Published:** 2011-06-17

**Authors:** Verena I Carrara, Celia Hogan, Cecilia De Pree, Francois Nosten, Rose McGready

**Affiliations:** 1 Shoklo Malaria Research Unit, Tak, Thailand; 2 Centre for Clinical Vaccinology and Tropical Medicine Churchill Hospital, Oxford, UK; 3 Faculty of Tropical Medicine, Mahidol University, Bangkok, Thailand

## Abstract

**Background:**

Maternal and infant health has been associated with maternal education level, which is highly associated with literacy. We aimed at estimating literacy rates among reproductive age women attending antenatal clinics in camps for refugees and in migrant clinics in Tak province, north-western Thailand, to determine whether illiteracy had an impact on birth outcomes.

**Methods:**

Three reading assessments were conducted using an identical method each time, in 1995-97, 2003 and 2008. Midwives chose at random one of four pre-set sentences. Each woman was asked to read aloud and scoring was based on a "pass/fail" system. Pregnancy outcomes were compared with maternal literacy rate.

**Results:**

Overall, 47% (1149/2424) of women were able to read. A significant improvement was observed among migrant (34% in 2003 *vs*. 46% in 2008, p = 0.01), but not refugee (47% in 1995-97, 49% in 2003, and 51% in 2008) women. Literate women were significantly more likely to be of non-Karen ethnicity, primigravidae, non-smokers, to remain free from malaria during pregnancy and to deliver in a health clinic. Significant improvements in pregnancy outcome (reductions in premature births, low birth weight newborns and neonatal death) between 1995-97 and 2003 were unrelated to literacy.

**Conclusions:**

Significant reductions in poor pregnancy outcome over time have not been driven by changes in literacy rates, which have remained low. Access to early diagnosis and treatment of malaria in this population, and delivery with skilled birth attendants, despite ongoing low literacy, appears to have played a significant role.

## Background

Low literacy skills have implications for health, whether in comprehending written instructions for taking medicines [1] or ability to understand one's medical condition [2]. Maternal and infant health has been associated with parental education levels, which can in part be measured by female literacy [3-5]. Maternal education level influences use of health services in Africa [6,7], Latin America [8], and Asia [9]. Studies have demonstrated improved pregnancy outcomes [10,11], reduced neonatal and infant mortality [12-15], and changes in reproductive health perception [16], with increasing levels of maternal education. Adult literacy programs have also demonstrated a reduction in infant mortality [17], and improvement in health-related knowledge [18]. In areas where a majority of women are illiterate or where schooling is poor or interrupted, innovative methods to deliver health messages and improve pregnancy outcomes need to be developed, as demonstrated recently in Nepal [19].

The latest World Health Organization estimate of maternal mortality for Burma was over 250/100,000 live-births; infant mortality was 50/1,000 live-births, of which, two-thirds occurred in rural areas, and less than half of pregnant women delivered with a skilled attendant [20]. The reported nationwide Burmese adult female literacy rate is high (89%) [21]. However, it fails to take into account geographical and ethnic differences. Ethno-linguistic minority native languages are not the language of education, nor are their scripts in the same alphabet, and there are over 100 different languages spoken in Burma [22]. Speakers of minority languages mostly live in rural areas where maternal and infant mortality are high.

On the eastern border of Burma prolonged armed conflict and economic difficulties have resulted in an influx of people to Thailand. This has contributed to the disruption of education in refugees and in migrants [23]. Children of migrant workers do not always have easy access to schooling or may drop out of school to help support the family; people arriving in the refugee camps, however, have the opportunity to enrol in an educational curriculum supported by non-governmental organizations [24]. Educational support and health care have been in place since the camps were established in the 1980s, and changes in health behaviour and improvements in education levels, in particular among the younger population, can be expected. In this manuscript we report literacy rates in pregnant women and explore the role of literacy in relation to poor pregnancy outcome on the Thai-Burmese border.

## Methods

### Objectives

This study aimed to evaluate the literacy rates of adult pregnant women attending antenatal care consultations in clinics for migrant populations, or in camps for displaced persons, and determine whether literacy had an impact on poor pregnancy outcome.

### Study area and survey participants

**Figure 1:**
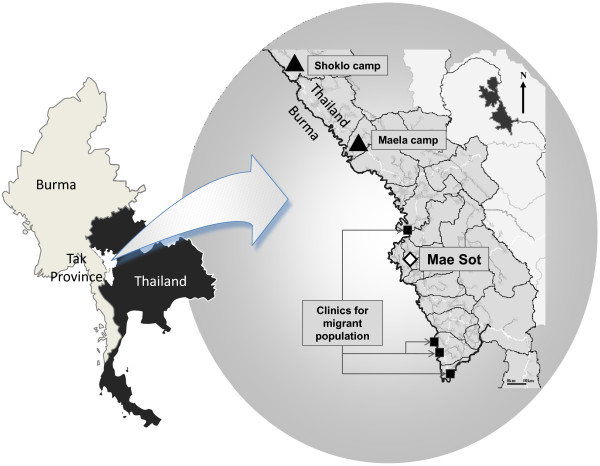
**Location of the different ANC clinics where literacy surveys among pregnant women were conducted during the 3 survey-periods, in the Thai province of Tak bordering Burma**. Location of SMRU clinics for camp population is represented by a triangle, location of clinics for migrant population by a square; Mae Sot is the main town of the Thai province of Tak bordering Burma

The ethno-linguistic origins of the population living along the Thai-Burmese border are diverse; the largest ethnic group, on both sides of the north-western border, is Sgaw Karen. Shoklo Malaria Research Unit (SMRU) provides free antenatal clinics (ANC) to camp and migrant populations. The camp population are families who fled armed conflict in Burma and settled in camps inside Thailand. The migrant population is composed of individuals and their families who are in search of work, often moving back and forth along the border (Figure 1). Minimum accepted age for marriage among the Karen community is 16 years; and although approximately 20% of pregnant women are teenagers, pregnancies occurring in the early teens are exceptional. Environment and living conditions have hardly changed over the years [25]; both refugee and migrant worker populations are from similarly deprived socio-economic backgrounds, although the population living in camps receives food and free access to medical care.

SMRU clinics are staffed by local health-workers. First antenatal consultation includes an obstetric and medical history and a detailed clinical examination for all women. Active antenatal screening for malaria and anaemia at antenatal clinics has been routine since 1986, because in this area transmission of malaria is low and seasonal but deleterious to pregnant women [26]. Women are encouraged to deliver with trained midwives in SMRU clinics where their newborn is weighed and examined.

### Eligibility for participation

Any woman attending ANC consultation (which is voluntary) during the period of the cross-sectional survey was eligible to participate.

### Enrolment criteria

There were no exclusion criteria, participation was voluntary and refusal to participate did not impact on the continuum of antenatal care. The first literacy assessment was part of a "malaria in pregnancy prevention" trial and was conducted at enrolment (April 1995-September 1997) [27]. The next two literacy assessments (October 2003 and April 2008) were part of planning IEC (Information, Education and Communication) materials on prevention of mother-to-child HIV transmission programs.

### Literacy assessment

Reading fluency is part of conceptual models developed to assess an individual's ability to comprehend messages or printed information [28]. Self-reported information on reading ability is subjective and often unreliable; education level attained does not always correlate with literacy skills and there are no standardized tests for assessing adult literacy in this multi-lingual setting [29]. We estimated women's literacy by their ability to read aloud a short sentence composed of words commonly used in local health messages. A 5-7 word health message sentence was typed in large font onto an A4 page mounted onto cardboard in three different languages: English, Burmese and Sgaw Karen. English was selected because it is taught in the camps and used by non-governmental teaching agencies supporting migrant communities. Cards were selected at random and the woman chose the language herself. The scoring was based on a "pass/fail" system: only women who could read aloud and fluently the complete sentence were reported as "can read". Women who read one language were asked if they could read another language and another card was taken at random and the exercise repeated. All three languages were scored individually. Women who mentioned their reading knowledge of a language not proposed on the cards were not tested formally, but were nonetheless scored as "can read another language". Fluency of reading was assessed by midwives able to speak, read and write in all three languages. During the 2008 survey, pregnant women were asked how many years they had attended school. If they said that they could read but had not attended school they were asked who taught them. Assessment was conducted face-to-face in a private area of the ANC.

### Pregnancy outcomes

Pregnancy outcomes were reported as miscarriage (pregnancy ending before 28 weeks of gestation), stillbirth, live-birth, or lost to follow up. Loss to follow-up was defined as discontinuation of antenatal care before the outcome of pregnancy was known (which occurs in this area due to population movement). Prematurity was defined as delivery before 37 weeks of gestation. Birth weight was valid if measured within the three first days of life, and low birth weight (LBW) was defined as a weight below 2500 g. Perinatal deaths included stillbirths and deaths of live born infants occurring within the first week of life, while neonatal deaths were deaths of live born infants occurring within 28 days of age. Gestational age was estimated by ultrasound at first consultation [30], by last menstrual period if known, or by Dubowitz gestational assessment scored between 6 and 72 hours after birth [31]. All congenital malformations were recorded. Minor malformations (i.e. skin tag) were reported as normal unless they were associated with another abnormality.

### Ethics statement

Entering and following ANC was voluntary, as was participation in the literacy assessments. The 1995-97 assessment was approved by the Ethical Review Committee of the Faculty of Tropical Medicine of Mahidol University, the Central Scientific Ethical Committee of Denmark and the Karen Refugee Committee [27]. Verbal consent was obtained during the next two assessments (2003 and 2008) as reading did not involve any risk for the pregnant woman.

Pregnancy records have been entered into an electronic data recording system since 1987. Ethical approval for retrospective analysis of pregnancy records was given by the Oxford Tropical Research Ethics Committee (OXTREC 28-09).

### Statistical Analysis

Data collected during the reading assessments were computerized separately (double-entered into Microsoft Excel spreadsheet), and linked to the core ANC database by the patient unique ID code number. Only variables necessary for the present analysis were selected. Data were analysed using SPSS for Windows™ (Version 14, SPSS Inc.). Continuous normally distributed data were described by their mean ± SD (i.e. age, estimated gestational age, birthweight), non-normally distributed data by their median and range (i.e. gravidity, parity). Percentages were given for categorical data, which were compared using the Chi-square test with Yates' continuity correction or Fisher's exact test. A paired t-test or Wilcoxon signed rank test were used to compare continuous variables. Factors associated with two variables, "ability to read" and "having a newborn of low birth weight", were evaluated by univariate analysis; two logistic regression models were created using "ability to read (yes/no)" and "LBW (yes/no)" as dependant variables. All factors with a p < 0.10 in univariate analysis were entered in their respective stepwise logistic regression model, and were included in the relevant tables. Adjusted odds ratios (AOR) were given with their 95% confidence interval.

## Results

A total of 2424 pregnant women agreed to be interviewed; 1965 were living in camps and 459 were from the migrant population.

### Demographic characteristics

Populations were similar in age, gravidity, and parity at each survey (Table 1).

**Table 1:** Demographic characteristics of pregnant women living in camp and in migrant populations, by survey-period

	1995-97	2003	2008
**Camp population**	**(*N *= 743)**	**(*N *= 547)**	**(*N *= 675)**

Age (year)^1^	25 ± 6 [15-44]	26 ± 7 [14-44]	26 ± 6 [15-48]
- Teenagers^2^	21% (157)	17% (95)	13% (86)
- 20-29 years^2^	53% (396)	50% (275)	56% (381)
- ≥ 30 years^2^	26% (190)	33% (177)	31% (208)
Primigravida^2^	27% (198)	22% (119)	26% (178)
Gravidity^3^	3 [1-14]	3 [1-11]	3 [1-17]
Parity^3^	1 [0-11]	2 [0-9]	1 [0-10]
Karen ethnicity^2^	85% (365)	88% (81)	81% (548)
Smokers^2^	41% (307)	30% (166)	21% (143)
Malaria in pregnancy^2^	25% (187)	7% (37)	7% (44)
Anaemia in pregnancy (Hct < 30%)^2^	71% (523)	61% (335)	28% (188)

**Migrant population**		**(*N *= 163)**	**(*N *= 296)**

Age (year)^1^		25 ± 7 [13-43]	27 ± 7 [15-46]
- Teenagers^2^		21% (34)	16% (46)
- 20-29 years^2^		50% (82)	55% (164)
- ≥ 30 years^2^		29% (47)	29% (86)
Primigravida^2^		28% (46)	28% (83)
Gravidity^3^		3 [1-12]	3 [1-12]
Parity^3^		1 [0-10]	1 [0-8]
Karen ethnicity^2^		70% (14)	64% (189)
Smokers^2^		35% (56)	26% (76)
Malaria in pregnancy^2^		50% (81)	37% (110)
Anaemia in pregnancy (Hct < 30%)^2^		57% (93)	47% (138)

Data are presented as mean ± SD [range] ^1^, as percentage (number) ^2^, or as median [range] ^3^

Over the span of 14 years there was a significant reduction of teenage pregnancies, women smoking, and having malaria or anaemia during pregnancy in the camp population. The same trends were observed in the migrant population, but were only significant for malaria and anaemia.

### Literacy assessment

Overall, 47% of pregnant women were able to read at least one language (*n *= 1149). Of those women who could read, 68% (*n *= 786) read one language, 24% (*n *= 278) read two, and 7% (*n *= 85) three or more. An equal number of women read Burmese or Sgaw Karen; however, Burmese was more likely to be read by the migrant population (86% (*n *= 165/192) *vs*. 60% (*n *= 577/957); p < 0.001), whilst Sgaw Karen was more frequently read by the camp population (71% (*n *= 676) *vs*. 19% (*n *= 37); p < 0.001). English was read by 8% (*n *= 91/1149) of the women with no significant difference between refugees and migrants. The ability to read another language was acknowledged by only 5.2% (*n *= 60) of women (17 reported reading Thai, 39 Poe Karen and 4 Arabic).

**Figure 2:**
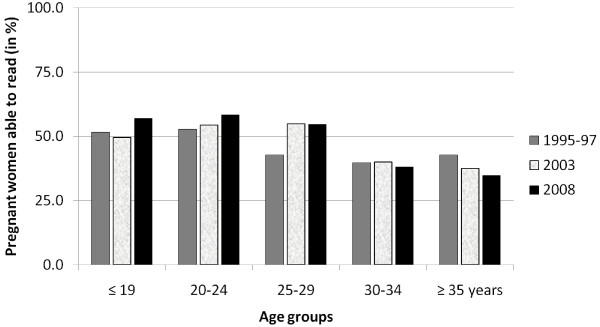
**Pregnant women attending ANC in the camps able to read (presented as percentage of total pregnant women assessed), by age-group and survey-period**.

**Figure 3:**
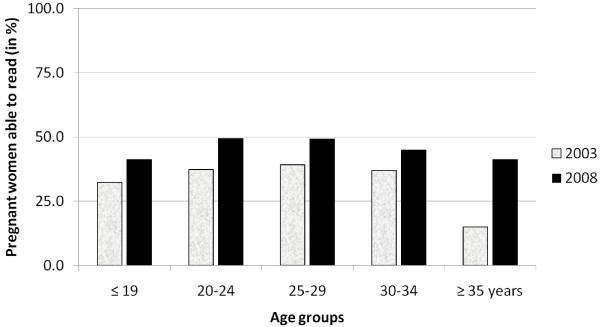
Pregnant women attending ANC in the clinics for migrant population (presented as percentage of total pregnant women assessed) able to read, by age-group and survey-period

Reading rate rose significantly among the migrant population, from 34% (*n *= 55) in 2003 to 46% (*n *= 137) in 2008, (p = 0.01), but not in the pregnant women living in the camps (47% (*n *= 350) in 1995-97, 49% (*n *= 266) in 2003 and 51% (*n *= 341) in 2008; p = 0.44). In 2008, the proportion of pregnant women able to read at least one language was identical in both populations. The small increase in proportion of teenage pregnant women able to read at least one language in either population was not significant (Figures 2 and 3). Reading two or more languages was more frequent among women living in the camps (35% (*n *= 335) *vs*. 15% (*n *= 29) in the migrants; p *<*0.001). In the 2008 interviews, the majority of women living in the camp who reported having attended school did so while still living in Burma or in Thai villages (56%, *n *= 193).

Pregnant women's demographic characteristics, habits or medical conditions that could potentially be associated with literacy were evaluated in univariate analysis and all of these are presented in table 2. All were significantly associated with literacy and were included in the regression model. After adjustment, three major health behaviour habits remained associated with the ability to read: less likely to smoke, less malaria during pregnancy and more likely to deliver in a health facility (Table 2).

**Table 2:** Maternal characteristics, habits and health risks associated with the ability to read (dependent variable) among pregnant women from migrant and camp populations (all 3 surveys combined, *n *= 2424)

**Variables examined**:	N	Ability to read % can read (n)	OR [95%CI]	AOR [95%CI]
< 30 yrs	1716	51% (876)	**0.6 [0.5-0.7]**	**0.3 [0.2-0.4]**
≥ 30 yrs	708	39% (273)	1.0	1.0

Primigravid	624	60% (374)	**0.5 [0.4-0.6]**	**0.6 [0.5-0.8]**
Multigravid	1800	43% (775)	1.0	1.0

Non-Karen	315	64% (200)	**0.5 [0.4-0.6]**	**0.7 [0.5-0.8]**
Karen	1197	45% (542)	1.0	1.0

Living in camp	1965	49% (957)	**0.8 [0.6-0.9]**	NS
Migrant workers	459	42% (192)	1.0	

Non-Smoking	1675	58% (966)	**0.2 [0.1-0.3]**	**0.3 [0.2-0.4]**
Smoking	748	24% (182)	1.0	1.0

No Malaria	1965	50% (975)	**0.6 [0.5-0.8]**	**0.7 [0.5-0.9]**
Malaria (+)	459	38% (174)	1.0	1.0

No Anaemia	1142	51% (583)	**0.8 [0.6-0.9]**	NS
Anaemia (+)	1277	44% (563)	1.0	

Clinic delivery	1228	53% (652)	**0.6 [0.5-0.7]**	**0.5 [0.4-0.7]**
Home delivery	1029	40% (414)	1.0	1.0

In bold, OR and AOR with a p < 0.05; all variables with p < 0.05 in univariate analysis were entered in the logistic regression model to obtain the AOR.

### Pregnancy outcomes

During the 1995-97 survey, most women delivered at home (80%). In 2003 and 2008 these proportions were inverted as 70% (*n *= 1090) of deliveries occurred in SMRU clinics. Outcomes were known for 93% (*n *= 2258) of pregnancies. There were 44 miscarriages and 26 stillbirths, 2% and 1% of pregnancy outcomes, respectively; these proportions were similar between the study-periods and the population groups. Twenty eight neonatal deaths were reported, 11 in 1995-97, 7 in 2003 and 10 in 2008, giving neonatal mortality rates of 16.1, 11.0 and 11.5 deaths per 1000 live births, respectively (p = 0.65). Risk of stillbirth, miscarriage, or neonatal death did not differ significantly between literate and illiterate women.

Characteristics of 2188 live newborns are presented in Table 3. There was a significant reduction in the proportion of premature birth and of LBW (p < 0.001 for both) in the camp population. In 2003 and 2008 the mean birth weight and proportion of LBW in both population groups were not significantly different.

**Table 3:** Birth outcomes, by population group and survey-period

	1995-97	2003	2008
**Camp population**			
*Known live births*	(*n *= 681)	(*n *= 515)	(*n *= 621)

EGA (wks, days)^1^	38.4 ± 1.8 [28.0-43.0]	39.0 ± 1.6 [28.5-42.5]	39.1 ± 1.5 [28.0-42.1]
Premature births	13% (87)	7% (35)	6% (35)
Male offspring	55% (371)	55% (282)	49% (303)

*Live births weighed in *≤ *3 days of life*	(*n *= 662)	(*n *= 498)	(*n *= 610)

Birth weight (g)^1^	2848 ± 504 [1007-4900]	2980 ± 443 [800-4500]	2968 ± 431 [1100-4220]
Low birth weight	18% (118)	11% (56)	11% (65)
Term LBW	10% (58)	9% (40)	8% (43)

**Migrant population**			
*Known live births*		(*n *= 124)	(*n *= 247)

EGA (wks, days)^1^		39.6 ± 2.0 [30.1-43.0]	39.0 ± 1.8 [28.6-43.4]
Premature births		7% (8)	8% (19)
Male offspring		55% (68)	53% (130)

*Live births weighed in *≤ *3 days of life*		(*n *= 56)	(*n *= 155)

Birth weight (g)^1^		2870 ± 466 [1050-4000]	2952 ± 492 [1250-4600]
Low birth weight		13% (7)	12% (18)
Term LBW		8% (4)	8% (11)

Data are presented as percentage (number) or as mean ± SD [range] ^1^

Maternal and newborn factors possibly associated with the risk of LBW are presented in table 4. Four maternal and two newborn factors were significantly associated with LBW and were entered into the regression model. Abnormal newborn, maternal malaria and place of delivery were also included in the model (p > 0.05 and < 0.1). After controlling for confounding factors, there was no association between literacy and the risk of LBW (Table 4).

**Table 4:** Maternal and newborn factors associated with low birth weight (LBW) (dependent variable) in newborns weighed within 3 days of birth (all 3 surveys combined, *n *= 1981)

Variables examined	N	Low Birth Weight	OR [95%CI]	AOR [95%CI]
**Maternal factors**		**% LBW (n)**		

Teenager	341	21% (70)	**1.9 [1.4-2.6]**	NS
Older	1640	12% (194)	1.0	

Primigravid	501	19% (97)	**1.9 [1.4-2.5]**	**1.9 [1.3-2.6]**
Multigravid	1480	11% (167)	1.0	1.0

Karen	1050	14% (144)	1.1[0.7-1.7]	NS
Non-Karen	252	12% (31)	1.0	

Migrant workers	211	12% (25)	0.9 [0.6-1.3]	NS
Living in camps	1770	14% (239)	1.0	

Illiterate	1010	15% (153)	**1.4 [1.1-1.8]**	NS
Literate	971	11% (111)	1.0	

Reading 1 language	321	11% (74)	1.0 [0.7-1.5]	NS
Reading >1 language	650	12% (37)	1.0	

Smoking	594	18% (107)	**1.7 [1.3-2.3]**	**1.9 [1.4-2.7]**
Non-Smoking	1387	11% (157)	1.0	1.0

Malaria (+)	318	17% (53)	*1.4 [1.0-1.9]*	NS
No Malaria	1663	13% (211)	1.0	

Anaemia (+)	1076	13% (144)	1.0 [0.8-1.3]	NS
No Anaemia	901	13% (120)	1.0	

Home delivery	839	15% (128)	*1.3 [1.0-1.7]*	NS
Clinic delivery	1142	12% (136)	1.0	

**Newborn factors**				

Prematurity	163	66% (108)	**20.9 [14.5-30.1]**	**20.3 [14.0-29.6]**
Term birth	1818	9% (156)	1.0	1.0

Female offspring	941	16% (148)	**1.5 [1.1-1.9]**	**1.8 [1.3-2.4]**
Male offspring	1040	11% (116)	1.0	1.0

Abnormal	30	27% (8)	*2.4 [1.0-5.5]*	NS
Normal	1951	13% (256)	1.0	

In bold, OR and AOR with p < 0.05, in italic with p ≥ 0.05 and < 0.1; all variables with p < 0.1 were entered in the logistic regression model to obtain the AOR

## Discussion

The literacy rate remains low amongst pregnant women in refugee camps or attending migrant ANC clinics on the Thai-Burmese border. Literacy rates have not improved amongst pregnant refugees despite non-governmental organisations supporting camp-based schools for the past 25 years, a finding already reported by Oh *et al*. [32]. These reported literacy rates are very similar to self-reported literacy in pregnant women in Eastern Burma [33]. However, some positive changes in birth outcomes have occurred in the almost 10-year period between the first (1995-97) and the second survey (2003), with a decrease in the proportion of premature births, LBW and a 40% reduction in neonatal mortality.

In this population illiteracy was not associated with any poor pregnancy outcomes. This does not preclude an indirect impact via a reduction over time in malaria in pregnancy (3 1/2 fold) and smoking (2 fold) and an increase in delivery with skilled birth attendants (2 1/2 fold). These three factors were associated with literacy in this population and all of them have been associated with birth outcomes previously [34-36]. The data here imply that the importance of illiteracy to poor pregnancy outcomes is relative to the severity of other contributing factors within a population. Access to early diagnosis and treatment of malaria in this population [37,38] and delivery with skilled birth attendants, despite ongoing low literacy, appears to have played a significant role. Similar findings have been described by Mullany *et al*. in Eastern Burma [33].

There were several limitations to this study. No measure of socio-economic status (SES) was included at each survey and improved SES may help explain the better outcomes. However, SMRU is an operational field based research unit and has not observed major changes in general living conditions, rations or health provision to these populations in over 20 years. The choice of literacy assessment was not ideal. Formal [39,40] and informal [41] assessments of adult literacy have been used to evaluate patients' health literacy for the past 20 years, although most have been validated in the most commonly used languages (i.e. English or Spanish). Few attempts have been made to develop simple instruments for literacy screening in Asian countries [42]. Our choice of using a 5-7 word sentence made-up of words used in health education messages was based on discussions with local health staff and with patients and the implications of being unable to read them, obvious to local health staff; as well as being easy and quick to perform given the time constraints of busy antenatal care consultations. Literacy and education level depend on ethnic origin and where people originate from in Burma; this information, particularly among migrant workers, was not systematically obtained. And whether this low literacy rate is a gender issue [43] or a neglected population issue remains uncertain as there has not been a comparison of male and female literacy in this population.

On a practical level, half of the pregnant population attending ANC is still unable to read and/or has had no formal education. Of those who can read, most read one of two languages (Burmese and Sgaw Karen). This has serious implications for any community-based interventions aimed at improving maternal and child health, the development and use of IEC health materials, explaining instructions on vitamins and medication and for obtaining informed consent [44]. Furthermore, many refugee women and their families will eventually resettle in foreign countries and their poor literacy skills may have consequences for their physical health and overall well being [45-47].

## Conclusions

The modest 12.5% and 1.9% reduction in illiteracy amongst reproductive age migrant and refugee women on the Thai Burmese border from 2003 to 2008 falls short of the goal set by the World Education Forum in Dakar in 2000, to reduce adult illiteracy by half by 2015. This study does not question the benefits of maternal literacy on pregnancy outcomes [10-15]. On the contrary, significant improvements in pregnancy outcome are possible without detectable improvements in female literacy. Health programmes must continue to address health needs in terms of access, acceptability and effectiveness.

## Abbreviations

ANC: antenatal clinic.

## Competing interests

The authors declare that they have no competing interests.

## Authors' contributions

RMG and FN conceptualized and designed the project. RMG, VIC, CH, CDP coordinated the cross-sectional surveys, interpreted the results, and drafted the manuscript, with FN. All authors revised the manuscript for intellectual content.

## Pre-publication history

The pre-publication history for this paper can be accessed here:

http://www.biomedcentral.com/1471-2393/11/45/prepub
